# Comparison of BMI and HbA1c changes before and during the COVID-19 pandemic in type 1 diabetes: a longitudinal population-based study

**DOI:** 10.1007/s40200-023-01316-z

**Published:** 2023-10-25

**Authors:** Marie Auzanneau, Dorothee M. Kieninger, Katharina Laubner, Christian Renner, Joaquina Mirza, Gerhard Däublin, Kirsten Praedicow, Holger Haberland, Claudia Steigleder-Schweiger, Bettina Gohlke, Angela Galler, Reinhard W. Holl, on behalf of the DPV Initiative

**Affiliations:** 1 Institute of Epidemiology and Medical Biometry, CAQM, Ulm University, Albert-Einstein-Allee 41, 89081 Ulm, Germany; 2 German Center for Diabetes Research (DZD), Munich, Neuherberg Germany; 3 Diabetes Division, Department of Paediatrics, Universitätsmedizin Johannes Gutenberg Universität Mainz, Mainz, Germany; 4 Division of Endocrinology and Diabetology, Department of Medicine II, Medical Center, University of Freiburg, Faculty of Medicine, University of Freiburg, Freiburg, Germany; 5 Pediatric Practice Deggendorf, Deggendorf, Germany; 6 Kinderkrankenhaus Amsterdamer Straße, Paediatric Diabetology, Klinik Für Kinder- Und Jugendmedizin, Kliniken Köln, Cologne, Germany; 7 Children’s Hospital Aurich, Aurich, Germany; 8 Clinic for Children and Adolescent Medicine, Diabetology and Endocrinology, Helios Clinical Centre Aue, Aue-Bad Schlema, Germany; 9 Paediatric Diabetology, Klinik Für Kinder- Und Jugendmedizin, Sana Klinikum Lichtenberg, Berlin, Germany; 10 Department of Paediatrics, Paracelsus Medical University, Salzburg, Austria; 11 Paediatric Endocrinology and Diabetology, University of Bonn, Bonn, Germany; 12 Charité, Universitätsmedizin Berlin, corporate member of Freie Universität Berlin Und Humboldt-Universität Zu Berlin, Sozialpädiatrisches Zentrum, Paediatric Endocrinology and Diabetology, Berlin, Germany

**Keywords:** COVID-19, Lockdown, BMI, HbA1c, Type 1 diabetes

## Abstract

**Purpose:**

To compare the changes in body weight and glycemic control before and during the COVID-19 pandemic in people with type 1 diabetes (T1D).

**Methods:**

In 47,065 individuals with T1D from the German Diabetes Prospective Follow-up Registry (DPV), we compared the adjusted mean changes in BMI-Z-scores and HbA1c as well as the distribution of individual changes between four periods from March 2018 to February 2022, by sex and age group (4- < 11, 11- < 16, 16–50 years).

**Results:**

At population level, the only significant pandemic effects were a slight increase in BMI Z-score in prepubertal children (girls: + 0.03 in the first COVID year vs. before, P < 0.01; boys: + 0.04, P < 0.01) as well as a stabilization of HbA1c in all subgroups or even improvement in women (− 0.08%, P < 0.01). At individual level, however, heterogeneity increased significantly (p < 0.01), especially in children. More prepubertal children gained weight (girls: 45% vs. 35% before COVID; boys: 39% vs. 33%). More pubertal girls lost weight (30% vs. 21%) and fewer gained weight (43% vs. 54%). More children had a decreasing HbA1c (prepubertal group: 29% vs. 22%; pubertal girls: 33% vs. 28%; pubertal boys: 32% vs. 25%) and fewer had increasing values. More women had stable HbA1c and fewer had increasing values (30% vs. 37%). In men, no significant changes were observed.

**Conclusion:**

This real-world analysis shows no detrimental consequences of the two first COVID years on weight and HbA1c in T1D on average, but reveals, beyond the mean trends, a greater variability at the individual level.

## Introduction

To date, only few studies have examined the impact of the COVID-19 pandemic and associated lockdown measures on people with type 1 diabetes (T1D). In particular, published evidence on the effect of social restrictions and stay-at-home orders on metabolic outcomes, such as BMI or HbA1c, is limited in this population. However, it is important to evaluate the potential consequences of the pandemic on diabetes management in people with T1D as a vulnerable group.

Results of studies investigating the effect of the lockdown on body weight in the general population are not necessarily transferrable to people living with T1D, and published results are inconsistent. Some analyses report weight gain associated with the pandemic, either in adults [1, 2] or in children [3–5], while other report an increase of cases of pediatric anorexia [6, 7], or weight changes in both directions [2]. A problem is that apart from rare exceptions [8, 9], the large majority of these reports are solely based on one difference (i.e., the comparison of two values, one before and one during the pandemic), and do not consider longer-term temporal trends including weight changes before the pandemic as control [2, 4]. Another frequent limitation is the report of population means, which hides the heterogeneity of the individual changes [3, 4]. In fact, mean estimates do not provide any information on the proportion of individuals with weight gain, weight loss, or stable weight, and how these proportions evolved over time. Most studies assessing the impact of the pandemic on weight and glycemic control in T1D present similar limitations [5, 10, 11].

Our aim in this analysis was therefore not only to compare mean trends over the years in the population, but also the distribution of the individual variations, in order to analyze whether the pandemic enlarged the heterogeneity of the individual changes or not. In addition, we sought to perform the analysis in the context of longer temporal trends, comparing changes during the pandemic with changes occurring before. We therefore compared changes in BMI-Z-score and HbA1c means and distributions from March 2018 to February 2022 in a large cohort of children and adults with T1D.

## Methods

### Study population

For this study, we used data from the multicenter Diabetes Prospective Follow-up Registry (DPV; Diabetes-Patienten-Verlaufsdokumentation), based at the University of Ulm, Germany. At the End of March 2022, 512 diabetes centers mainly located in Germany and Austria have been participating in this registry. All of them prospectively collect and document data on diabetes treatment and outcomes in the standardized DPV electronic health record, and transmit every six months pseudo-anonymized data to the University of Ulm. After plausibility checks, the University of Ulm reports inconsistent data back to the centers for correction and validation. Afterwards, anonymized data is used for benchmarking and patient-centered analyses. Data analysis is approved by the Ethics Committee of the Medical Faculty of the University of Ulm (Number 314/21). At the End of March 2022, 643,759 individuals with any type of diabetes have been documented in the DPV registry, with 156,058 individuals classified as T1D. In this longitudinal study, we included patients with T1D (age at diagnosis ≥ 6 months; diabetes duration ≥ 3 months), and residence in Germany. In addition, we decided to restrict the analysis to people aged between 4 and 50 years to form homogeneous age groups (excluding older patients who have more frequently long-term complications and other comorbidities) and to consider only people in school or working age, whom we expect to have been more affected by school closures and stay-at-home orders. We then analyzed all visits between March 2018 and February 2022.

### Variables

In the DPV database, the definition of T1D is based on a physician’s diagnosis according to the ISPAD or ADA guidelines [12, 13]. The BMI-Z-score is defined according to the reference values of the German Association for the Study of Obesity (Deutsche Adipositas Gesellschaft, DAG, Arbeitsgemeinschaft Adipositas [AGA]) for 0- to 79-year-old [14]. To adjust for differences between laboratories, HbA1c values were standardized to the reference range of the Diabetes Control and Complications Trial (DCCT) (4.05–6.05% [20.7–42.6 mmol/mol]) using the multiple of the mean method [15].

We adjusted for migration background and socioeconomic situation which are both known to influence weight and glycemic control in T1D, also in Germany [16, 17]. Migration background is defined as place of birth outside Germany for the patient or at least for one parent. To take the socioeconomic situation of the patients into consideration, we used the German Index of Socioeconomic Deprivation of the year 2012 (GISD_2012_) [18], which is open for research at the data repository of the German GESIS Leibniz-Institute for the Social Sciences (https://doi.org/10.7802/1460). The GISD_2012_ encompasses aggregated data on education, occupation, and income at the regional level, following a methodology described previously [18]. Individuals were assigned to districts and consequently to GISD_2012_ quintiles using the five-digit postcode of their residence. Districts were categorized into deprivation quintiles, from Q1 (lowest deprivation) to Q5 (highest deprivation). Individuals without postcode were excluded from the analysis (n = 709), since they could not be assigned to a district and therefore to a deprivation quintile.

### Statistical analysis

We defined four time-periods: time 1 from March 2018 to February 2019, time 2 from March 2019 to February 2020, time 3 from March 2020 to February 2021, time 4 from March 2021 to February 2022. For each patient, longitudinal data (age, diabetes duration, BMI-Z-score, HbA1c) was aggregated as median for the respective time-period. Median age per period of time was categorized into three groups, roughly related to pubertal status: 4‒ < 11 years (prepubertal), 11‒ < 16 years (pubertal), and 16‒ ≤ 50 years (postpubertal and adult). Over the four periods of time from March 2018 to February 2022, 76,6% of the individuals (n = 36,032) remained in the same age category, 9.4% of the individuals (n = 4,443) transited from the prepubertal to the pubertal group, and 14.0% of the individuals (n = 6,590) transited from the pubertal to the postpubertal group. We took the age group of the first period of time into account to perform the analyzes stratified by sex and age group.

To investigate the mean changes in each subgroup before COVID, as well as in the first and second COVID year, mean BMI-Z-Score and HbA1c were estimated in each time period (time 1 to time 4) using linear regression models, adjusted for diabetes duration, migration background, and quintile of socioeconomic deprivation, with repeated measurements within individuals (patient as random effect). Then, the mean differences between the estimates of two periods were analyzed using respective models.

To analyze the distribution of the individual changes, we calculated for each patient the BMI-Z-Score- and HbA1c change between the median values in times 2 and 1 (change before COVID), times 3 and 2 (change in the first COVID year), and times 4 and 3 (change in the second COVID year). In addition, we calculated in each subgroup and time period the proportion of individuals with weight loss (BMI-Z-score difference <  − 0.1), stable weight (BMI-Z-score difference between − 0.1 and + 0.1), and weight gain (BMI-Z-score difference >  + 0.1). Similarly, we calculated the proportion of individuals with decreasing, stable, and increasing HbA1c before COVID and during the first two COVID years (HbA1c difference <  − 0.2; between − 0.2 and + 0.2, and >  + 0.2 respectively).

Unadjusted patient characteristics are presented as median with lower and upper quartile (Q1-Q3) for continuous variables, or as proportion for variables with binomial distribution. Wilcoxon tests and X^2^ tests, adjusted for multiple comparisons according to the Holm-Bonferroni stepdown procedure, were used to compare these characteristics between males and females. Results of regression analyses are presented as adjusted estimates with their 95% confidence intervals (95% CI). P-values were adjusted for multiple comparisons according to the Tukey–Kramer procedure. A p-value < 0.01 (two-sided) was considered statistically significant. Statistical analysis was performed using SAS 9.4, built TS1M7 on a Windows server 2019 mainframe (SAS Institute, Cary, NC).

## Results

A total of 47,065 children and adults met the inclusion criteria (diagnosis of T1D for at least three months, n = 146,059; age between 4 and 50 years, n = 124.080; visits recorded between March 2018 and March 2022, n = 53,443; residence in Germany with postcode available, n = 47,065). Characteristics of the study population stratified by sex and age group are presented in Table 1. In the prepubertal group, none of the observed characteristics differed significantly between girls and boys. From the age of 11 and above, girls had a longer diabetes duration compared to boys of the same age group, as well as a higher BMI-Z-score (Table 1). Median HbA1c was slightly higher in pubertal females compared to males (Table 1).

**Table 1:** Characteristics of the study population

Study population (n = 47,065)				
By age group		By sex		
		Female (n = 22,087)	Male (n = 24,978)	P-values
Prepubertal: 4- < 11 years (n = 13,607)	Age, years (median, Q1-Q3) Diabetes duration, years (median, Q1-Q3) Migration background, (%) BMI-Z-score, AGA, (median, Q1-Q3) HbA1c, % (median, Q1-Q3)	8.0 (5.9–9.6) 1.3 (0.6–3.3) 30.1 0.25 (-0.32–0.85) 6.97 (6.42–7.52)	7.9 (5.8–9.6) 1.4 (0.6–3.4) 30.5 0.27 (-0.31–0.89) 6.90 (6.37–7.50)	0.39 0.04 0.65 0.53 0.05
Pubertal: 11- < 16 years (n = 15,652)	Age, years (median, Q1-Q3) Diabetes duration, years (median, Q1-Q3) Migration background, n (%) BMI-Z-score, AGA, (median, Q1-Q3) HbA1c, % (median, Q1-Q3)	13.5 (12.3–14.7) 3.9 (0.9–7.4) 28.3 0.47 (-0.20–1.16) 7.20 (6.54–7.92)	13.7 (12.4–14.8) 3.0 (0.7–6.9) 27.5 0.20 (-0.48–0.93) 7.12 (6.43–7.88)	< 0.001 < 0.001 0.65 < 0.001 < 0.001
Postpubertal and adults: 16–50 years (n = 17,806)	Age, years (median, Q1-Q3)	20.6 (17.5–34.3)	20.2 (17.4–35.1)	0.40
	Diabetes duration, years (median, Q1-Q3)	9.6 (4.8–15.9)	8.4 (3.6–14.7)	< 0.001
	Migration background, n (%)	26.4	24.5	0.25
	BMI-Z-score, AGA, (median, Q1-Q3)	0.46 (-0.33–1.20)	0.03 (-0.80–0.87)	< 0.001
	HbA1c, % (median, Q1-Q3)	7.43 (6.64–8.48)	7.47 (6.64–8.67)	0.12

Unadjusted data. Q1-Q3: lower–upper quartile

### BMI-Z-score

- ***Adjusted mean BMI-Z-score trends before and during the pandemic***

**Fig. 1:**
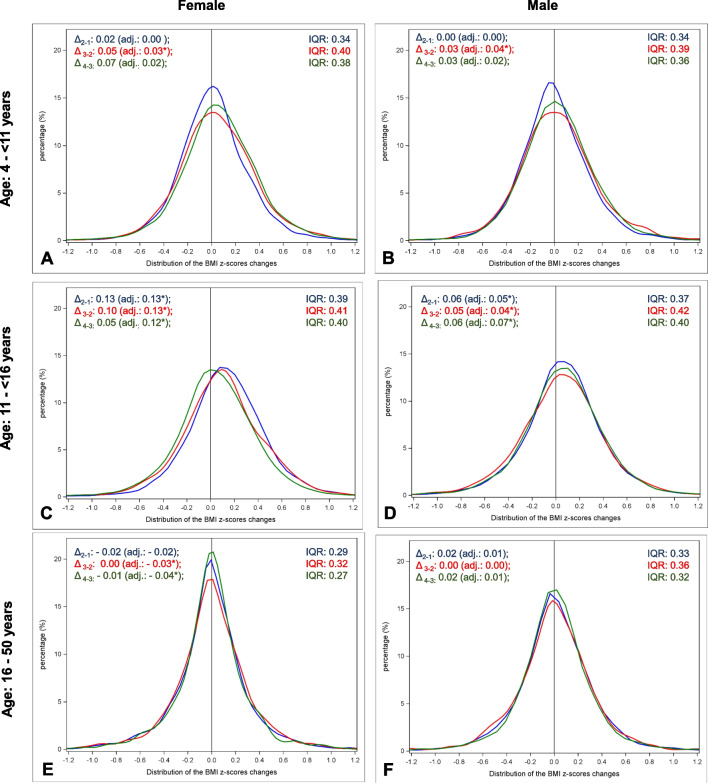
Distribution of BMI-Z-scores changes before and during the COVID-19 pandemic in patients with type-1-diabetes, stratified by sex and age group. Δ_2-1_, Δ_3-2_ or Δ_4-3:_ mean change between time 2 (March 2019-February 2020) and time 1 (March 2018-February 2019), time 3 (March 2020-February 2021) and time 2, time 4 (March 2021-February 2022) and time 3. adj.: Estimated mean change adjusted for diabetes duration, migration background, and socioeconomic deprivation, and repeated measurements within patients (random effect). *significant (P < 0.01), adjusted for multiple comparisons according to the Tukey–Kramer procedure). IQR: interquartile range. Distribution of individual BMI Z-score changes: Before COVID (time 2 – time 1) in blue; With COVID first year (time 3 – time 2) in red, With COVID second year (time 4-time 3) in green

In all subgroups, the adjusted BMI-Z-score changes were similar during the two COVID years compared to the year before, except in prepubertal children (Fig. 1). In this age group, we observed a slight but significant BMI-Z-score increase in the first COVID year compared to the year before, without significant difference by sex (adjusted mean change in the first COVID year vs. before: + 0.03 [P < 0.01] vs. 0.00 [P = 0.99] in girls; + 0.04 [P < 0.01] vs. 0.00 [P = 0.49] in boys; comparison by sex: p = 0.07). In the other age groups, the BMI trends before COVID continue to evolve similarly during the two years of the pandemic: the BMI-Z-score continue to increase in pubertal girls, and to a lesser extent in boys (Fig. 1). The BMI-Z-score continued to decrease slightly in females aged 16–50 years and remained stable during the whole observation period in males of the same age group (Fig. 1).

- ***Distribution of the individual BMI-Z-score changes before and during the pandemic***

Regarding the distribution of the individual BMI-Z-scores changes, more heterogeneity was observed during the first COVID year (Interquartile range [IQR] wider resulting from a lower proportion of individuals with stable weight) compared to the year before (Fig. 1). This was especially the case in the prepubertal group, in both girls and boys, as well as in pubertal boys (Fig. 1). In the second COVID year, the heterogeneity declined slightly in all children aged < 16 years, and to a greater extent in postpubertal individuals and adults. In the prepubertal group, the greater heterogeneity during the two COVID years was characterized by a higher proportion of children with weight gain (45% in the second COVID year vs. 35% before COVID in girls and 39% vs. 33% in boys, both P < 0.001, Table 2). In the pubertal group, the proportions of boys with stable weight, weight loss, and weight gain in the three time periods remained similar (Table 2). By contrast, the proportion of pubertal girls with weight loss increased considerably in the second COVID year compared to the year before (30% vs. 21%), whereas those with weight gain decreased in a similar proportion (43% vs. 54%, both differences: P < 0.001, Table 2). Observation of Fig. 1C conforms to these results: although the adjusted mean changes remained similar over the years, the unadjusted mean changes decreased in the second COVID year (green curve shifted to the left). In the group aged 16–50 years, changes were not significant (Table 2).

**Table 2:** Proportion of individuals with weight loss, stable weight, and weight gain before COVID and during the first two COVID years, stratified by age group and sex

Age group	Sex	Time period*	With weight loss** (%)	P-value***	With stable weight** (%)	P-value***	With weight gain** (%)	P-value***
Prepubertal: 4- < 11 years	Female	Before COVID	33.01	< 0.001	31.98	0.004	35.02	< 0.001
		With COVID first year	31.31		26.93		41.76	
		With COVID second year	26.98		28.20		44.82	
	Male	Before COVID	34.63	0.329	32.28	0.050	33.09	< 0.001
		With COVID first year	34.36		27.19		38.45	
		With COVID second year	32.17		29.25		38.58	
Pubertal: 11- < 16 years	Female	Before COVID	20.73	< 0.001	25.31	1.000	53.96	< 0.001
		With COVID first year	25.76		25.22		49.01	
		With COVID second year	30.17		26.71		43.12	
	Male	Before COVID	27.00	1.000	28.11	0.477	44.89	1.000
		With COVID first year	30.94		24.70		44.36	
		With COVID second year	28.16		26.26		45.58	
Postpubertal and adults: 16–50 years	Female	Before COVID	31.19	1.000	37.61	1.000	31.19	1.000
		With COVID first year	31.77		35.24		32.99	
		With COVID second year	30.56		39.31		30.12	
	Male	Before COVID	32.12	1.000	32.33	1.000	35.55	1.000
		With COVID first year	33.41		30.92		35.68	
		With COVID second year	31.17		33.73		35.09	

^*^ Before COVID: changes between time 2 (March 2019-February 2020) and time 1 (March 2018-February 2019); With COVID first year: changes between time 3 (March 2020-February 2021) and time 2; With COVID second year: changes between time 4 (March 2021-February 2022) and time 3

^**^ Weight loss: BMI-Z-score difference < -0.1; stable weight BMI-Z-score difference between -0.1 and + 0.1; Weight gain: BMI-Z-score difference >  + 0.1

^***^ Chi^2^-Test for comparison before COVID vs. COVID second year. P-values adjusted for multiple tests according to the Bonferroni procedure

### HbA1c

- ***Adjusted mean HbA1c trends before and during the pandemic***

**Fig. 2:**
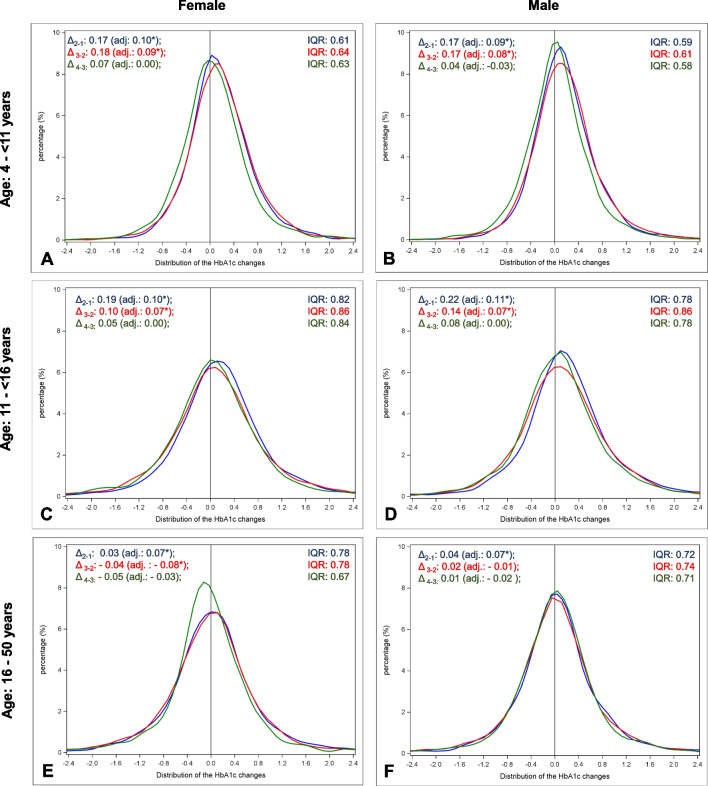
Distribution of HbA1c changes before and during the COVID-19 pandemic in patients with type-1-diabetes, stratified by sex and age group. Δ_2-1_, Δ_3-2_ or Δ_4-3:_ mean change between time 2 (March 2019-February 2020) and time 1 (March 2018-February 2019), time 3 (March 2020-February 2021) and time 2, time 4 (March 2021-February 2022) and time 3. adj.: Estimated mean change adjusted for diabetes duration, migration background, and socioeconomic deprivation, and repeated measurements within patients (random effect). *significant (P < 0.01), adjusted for multiple comparisons according to the Tukey–Kramer procedure). IQR: interquartile range. Distribution of individual HbA1c changes: Before COVID (time 2 – time 1) in blue; With COVID first year (time 3 – time 2) in red, With COVID second year (time 4-time 3) in green

The adjusted mean HbA1c trend changed with the second COVID year compared to the years before in all children < 16 years (Fig. 2): mean HbA1c values increased before and during the first COVID year, and then stabilized. In older subjects, the HbA1c trend changed similarly but already in the first COVID year (Fig. 2): after an increase before COVID (+ 0.07% [+ 0.02; + 0.12] in females and + 0.07% [+ 0.02; + 0.11] in males, both P < 0.01), mean HbA1c then stabilized (males) or even decreased (females: − 0.08% [− 0.13; − 0.03], P < 0.01).

- ***Distribution of the individual HbA1c-changes before and during the pandemic***

Except in women > 16 years, more individual HbA1c changes were observed in all subgroups during the first COVID year (IQR wider) compared to the year before (Fig. 2). This was especially the case in pubertal boys. However, this greater heterogeneity decreased in the second COVID year (Fig. 2). After two COVID years, the lower proportion of children with increasing HbA1c was replaced by a higher number with decreasing HbA1c (29% vs. 22% in both girls and boys in the prepubertal group, 33% vs. 28% in pubertal girls, and 32% vs. 25% in pubertal boys, all P < 0.001, Table 3). In women, we observed more homogeneity: the proportion with increasing HbA1c fell from 37 to 30% and the proportion with stable HbA1c grew from 28 to 33%, both P < 0.001. In men, the distribution of the changes remained similar before and during the pandemic (Table 3).

**Table 3:** Proportion of individuals with decreasing, stable, and increasing HbA1c before COVID and during the first two COVID years, stratified by age group and sex

Age group	Sex	Time period*	With decreasing HbA1c** (%)	P-value***	With stable HbA1c** (%)	P-value***	With increasing HbA1c** (%)	P-value***
Prepubertal: 4- < 11 years	Female	Before COVID	21.68	< 0.001	34.23	1.000	44.09	< 0.001
		With COVID first year	21.72		32.83		45.45	
		With COVID second year	29.10		34.37		36.53	
	Male	Before COVID	21.80	< 0.001	35.52	1.000	42.68	< 0.001
		With COVID first year	22.26		33.11		44.62	
		With COVID second year	29.13		36.92		33.95	
Pubertal: 11- < 16 years	Female	Before COVID	27.60	< 0.001	25.59	1.000	46.81	< 0.001
		With COVID first year	32.62		25.26		42.13	
		With COVID second year	33.42		26.62		39.96	
	Male	Before COVID	25.35	< 0.001	27.11	1.000	47.55	< 0.001
		With COVID first year	30.97		24.85		44.18	
		With COVID second year	32.08		27.93		39.99	
Postpubertal and adults: 16–50 years	Female	Before COVID	35.17	1.000	27.76	0.001	37.07	< 0.001
		With COVID first year	36.30		28.22		35.47	
		With COVID second year	36.47		33.19		30.33	
	Male	Before COVID	32.56	1.000	31.50	1.000	35.94	1.000
		With COVID first year	32.89		31.84		35.27	
		With COVID second year	32.55		31.89		35.56	

^*^ Before COVID: changes between time 2 (March 2019-February 2020) and time 1 (March 2018-February 2019),

With COVID first year: changes between time 3 (March 2020-February 2021) and time 2,

With COVID second year: changes between time 4 (March 2021-February 2022) and time 3

^**^ Decreasing HbA1c: HbA1c difference < -0.2; stable HbA1c: HbA1c difference between -0.2 and + 0.2; Increasing HbA1c: HbA1c difference >  + 0.2

^***^ Chi^2^-Test for comparison before COVID vs. COVID second year. P-values adjusted for multiple tests according to the Bonferroni procedure

## Discussion

This longitudinal analysis of registry-based data documented between 2018 and 2022 reveals that the two pandemic years were not associated with systematic detrimental consequences on weight and glycemic control in our population with T1D. However, the individual responses of people with T1D to the COVID-19 pandemic, in terms of BMI or HbA1c-changes, differed widely.

Considering only the adjusted mean changes, BMI trends were not affected by the pandemic, except a slight increase in prepubertal children. Nevertheless, beyond the mean, it appears clearly that the first year of the pandemic has been accompanied by more individuals with BMI increases or decreases, and that this greater heterogeneity particularly affected children and adolescents < 16 years. With the second COVID year, the proportion of individuals with stable BMI increased again, but we observed a larger proportion of prepubertal children with weight gain, as well as a larger proportion of pubertal girls with weight loss. While increasing before COVID, mean HbA1c trends stabilized during the pandemic in all subgroups or even improved in women. At individual level, no significant change was observed in adult men, but in all other subgroups, the proportion of individuals with increasing HbA1c decreased.

Whereas some studies performed in the general population have found a greater BMI increase in all children during the pandemic compared to previous years [3, 5], other found that this was more pronounced or, like in our results, restricted to prepubertal children [8, 19]. In general, the accelerated increase in BMI in children due to the pandemic has often been explained as a consequence of reduced physical activity and altered eating habits due to the widespread closure of schools and leisure or sport facilities during the lockdown [3]. In line with these studies, we found a higher proportion of children with weight gain, but the average excess BMI-Z-score gain related to the pandemic in this age group was very small. It is possible that the impact of the COVID-lockdown on children’s weight development has been attenuated in the presence of diabetes. This disease being part of well-documented risk factors for worse COVID outcomes, parents of children with this chronic condition could have paid particularly attention to alimentation and/or to physical activities during the pandemic [20]. In addition, working from home, which was widespread during the lockdown, may have made it easier to prepare healthy meals, and improved parental supervision. Moreover, children with diabetes and their parents may have benefited from more frequent medical consultations and health advices than people without chronic disorder. Thus, T1D could have constituted a moderating factor against weight gain in this time period when compared to the general population.

Regarding older children and adults, longitudinal studies in the general population which have taken a pre-pandemic control period into consideration indicate that BMI trends were not modified by the pandemic [9, 10, 21]. Our findings in adolescents and adults with T1D confirm these results. In pubertal girls however, even if the proportion with weight gain was still greater that the proportion with weight loss after two years pandemic, about one third more have lost weight compared to the year before. This aspect is not apparent when only the adjusted mean values are evaluated. These results are in line with many other studies indicating more cases of eating disorders, in particular in young women, in conjunction with the pandemic [6, 7, 22].

Our findings indicate a stabilization or even an improvement of glycemic control in the second COVID-year in both children and adults with T1D. During the pandemic, the proportion of individuals with increasing HbA1c fell in nearly all subgroups. Few studies found no significant changes in children [11] or only very slight changes in adults [10], but a greater number of analyses [23, 24], including three systematic reviews [5, 25, 26], found better glycemic control, in line with our results. To explain the possible positive effect of the lockdown on glycemic control in T1D, several hypotheses have been formulated [5]. In particular, the stay-at-home orders may have been associated with more time, not only for diabetes management, but also for self-care, healthy meals and exercise. Moreover, the lockdown could have enabled a more predictable daily routine, with more regular meal and sleep times [27]. Last but not least, the increasing use of telemedicine during the pandemic [28] combined with a widespread use of diabetes technology (in particular, continuous glucose monitoring [CGM] and automated insulin delivery [AID]) in T1D seems to have contribute to an effective diabetes management despite lockdown [11].

A limitation of this study is that we did not take the use of diabetes technology into account, although diabetes technology and CGM in particular is associated with improved glycemic control [29]. For example, a more frequent use of CGM during the pandemic could have improved glycemic outcomes. Another bias could have resulted from potential differences in frequency of weight and HbA1c measurements during the pandemic compared to the period before. However, a recent study from Germany indicates that access to healthcare did not change considerably during the pandemic for children and adolescents with T1D [30]. For our study population, BMI and HbA1c were documented only slightly less frequently during the pandemic compared to the pre-pandemic period (in mean: 3.6 instead of 4.2 times per year for BMI, 3.1 instead of 3.4 times per year for HbA1c). Nevertheless, a possible selection bias could have affected the results, if individuals with worse glycemic control had fewer medical visits or less health care utilization during the pandemic than those with better glycemic control. Inversely, one could consider that especially individuals with worse glycemic control were more prone to visit their diabetes center during the pandemic than other individuals with better health outcomes. A strength of this longitudinal analysis is the use of repeated measurements over four years, which allows the comparison of trends between the COVID and the pre-COVID periods. In addition, real world evidence could have been provided by the use of a nationwide registry comprising about 90% of children and adolescents and about 30% of adults with T1D in Germany. Moreover, the report of the distribution of individual weight and HbA1c changes over the years provides useful information to understand the real impact of two years COVID in people living with T1D.

To summarize, this longitudinal analysis contributes to reduce the concerns over potential detrimental impact of the COVID pandemic on health outcomes of individuals with T1D. First, the pandemic seems to have been associated with perturbations or instability in terms of weight and glycemic control at the individual level, but eventually, in the second COVID year, these variations have been significantly reduced in all subgroups. It is possible that most individuals progressively adapted their lifestyle to the pandemic situation with its lockdown restrictions, and that the psychological impact of the pandemic consequently reduced over time. In addition, in our large population with T1D, the pandemic was not associated with clinically relevant changes in BMI trends, but with a slight improvement in the HbA1c trend. Stay-at-home orders combined with the fear of adverse COVID outcomes in individuals with diabetes may have encouraged a heathier lifestyle [5]. In addition, the development of telemedicine [28] associated with the increasing use of diabetes technology may have played a positive role during the pandemic.

In conclusion, even if the COVID pandemic did not affect weight and glycemic control negatively in individuals with T1D on average, our data indicates greater variation in some subgroups, in particular children and adolescents. Since weight and glycemic variability are associated with adverse diabetes-related outcomes [31, 32], it is important to pay attention to these changes to improve the care of vulnerable groups in the future.

## Data Availability

Aggregated data might be made available upon reasonable request via email to the senior author reinhard.holl@uni-ulm.de.
